# Supplementary material for: Associations between Hypertension, Treatment, and Cognitive Function in the Irish Longitudinal Study on Ageing
Source: J Clin Med. 2020 Nov 20;9(11):3735. doi: 10.3390/jcm9113735 (PMC7699900; doi:10.3390/jcm9113735)
Supplement: Supplementary file 1 [file jcm-09-03735-s001.pdf]

Supplement

	Page
**Hypertensive status**	
Table 1. Multivariate regression results of the effects of hypertensive status (H1, H2, H3) on MMSE controlling for age, education and other socio-demographic and health behaviours (full model).	2
Table 2. Multivariate regression results of the effects of hypertensive status (H1, H2, H3) on MoCA controlling for age, education and other socio-demographic and health behaviours (full model).	3
Table 3. Multivariate regression results of the effects of hypertensive status (H1, H2, H3) on CTT-1 controlling for age, education and other socio-demographic and health behaviours (full model).	4
Table 4. Multivariate regression results of the effects of hypertensive status (H1, H2, H3) on CTT-2 controlling for age, education and other socio-demographic and health behaviours (full model).	5
**Medication categories**	
Table 5. Multivariate regression results of the effects of medication categories (BB, CB, ACE) on MMSE controlling for age, education and other socio-demographic and health behaviours (full model).	6
Table 6. Multivariate regression results of the effects of medication categories (BB, CB, ACE) on MoCA controlling for age, education and other socio-demographic and health behaviours (full model).	7
Table 7. Multivariate regression results of the effects of medication categories (BB, CB, ACE) on CTT-1 controlling for age, education and other socio-demographic and health behaviours (full model).	8
Table 8. Multivariate regression results of the effects of medication categories (BB, CB, ACE) on CTT-2 controlling for age, education and other socio-demographic and health behaviours (full model).	9
**Medication status**	
Table 9. Multivariate regression results of the effects of medication status (monotherapy and combination therapy) on MMSE controlling for age, education and other socio-demographic and health behaviours (full model).	10
Table 10. Multivariate regression results of the effects of medication status (monotherapy and combination therapy) on MoCA controlling for age, education and other socio-demographic and health behaviours (full model).	11
Table 11. Multivariate regression results of the effects of medication status (monotherapy and combination therapy) on CTT-1 controlling for age, education and other socio-demographic and health behaviours (full model).	12
Table 12. Multivariate regression results of the effects of medication status (monotherapy and combination therapy) on CTT-2 controlling for age, education and other socio-demographic and health behaviours (full model).	13

Table 1. Multivariate regression results of the effects of hypertensive status (H1, H2, H3) on MMSE controlling for age, education and other socio-demographic and health behaviours (full model).

	Wave 1				Wave 3				Longitudinal			
**Source**	**β**	**SE**	**p-value**	**ηp2**	**β**	**SE**	**p-value**	**ηp2**	**β**	**SE**	**p-value**	**ηp2**
**MMSE**												
Hypertensive status				<0.01				<0.01				<0.01
H2	-0.02	0.09	0.350		-0.01	0.08	0.602		-0.03	0.10	0.139	
H3	-0.02	0.08	0.301		0.01	0.06	0.622		-0.02	0.07	0.237	
Age	-0.25	0.01	<0.0001	0.04	-0.26	0.01	<0.0001	0.04	-0.20	0.01	<0.0001	0.02
Education				0.09				0.05				0.01
Secondary education	0.27	0.08	<0.0001		0.23	0.08	<0.0001		0.09	0.10	0.002	
Higher education	0.38	0.08	<0.0001		0.27	0.08	<0.0001		0.12	0.10	<0.0001	
Sex				<0.01				0.01				<0.01
Female	0.05	0.05	<0.0001		0.08	0.05	<0.0001		0.02	0.06	0.186	
Employment				<0.01				0.01				<0.01
Retired	0.04	0.08	0.033		0.02	0.06	0.353		0.01	0.08	0.858	
Other	-0.04	0.07	0.012		-0.09	0.07	<0.0001		-0.03	0.07	0.051	
Antidepressants				<0.01				0.01				<0.01
Taking antidepressants	-0.05	0.13	0.001		-0.08	0.11	<0.0001		-0.02	0.13	0.253	
Smoking status				<0.01				0.01				<0.01
Past smoker	0.02	0.06	0.157		0.01	0.05	0.532		-0.01	0.06	0.472	
Current smoker	-0.05	0.08	0.001		-0.05	0.09	0.003		-0.01	0.09	0.025	
Alcohol problem				<0.01				<0.01				<0.01
Yes	0.02	0.07	0.033		0.02	0.07	0.264		-0.01	0.07	0.735	<0.01
Moderate physical activity				<0.01				<0.01				
<5 times/week	0.01	0.06	0.601		0.04	0.05	<0.0001		0.01	0.06	0.338	<0.01
Diabetes	-0.02	0.14	0.321	<0.01				<0.01				<0.01
Yes	0.02	0.003	0.440		-0.02	0.10	0.169		-0.02	0.12	0.215	
Systolic blood pressure	0.01	0.004	0.579	<0.01	0.05	0.01	0.045	<0.01	-001	0.01	0.822	<0.01
Diastolic blood pressure				<0.01	-0.05	0.01	0.055	<0.01	0.04	0.01	0.102	<0.01
Baseline MMSE									0.36	0.05	<0.0001	0.13
**Overall model**	F (16, 5205) =63.03, p<0.0001, η2 =0.20				F (16, 4510) =38.14, p<0.0001, η2 =0.16				F (17, 3104) =33.90, p<0.0001, η2 =0.27			

Comparison group: hypertensive status = H1 (no hypertension), education = primary education, sex = male, employment = currently employed, antidepressant = not taking antidepressants, smoking status = non-smoker, alcohol problem = no alcohol problem, moderate physical activity = ≥5 times/week, diabetes = no diabetes.

Note: Hypertensive status consists of H1 (no hypertension), H2 (hypertension without medication), H3 (hypertensive with medication). Longitudinally, the dependent outcome (MMSE) at W3 was controlled for its baseline score.

Table 2. Multivariate regression results of the effects of hypertensive status (H1, H2, H3) on MoCA controlling for age, education and other socio-demographic and health behaviours (full model).

	Wave 1				Wave 3				Longitudinal			
**Source**	**β**	**SE**	**p-value**	**ηp2**	**β**	**SE**	**p-value**	**ηp2**	**β**	**SE**	**p-value**	**ηp2**
**MOCA**												
Hypertensive status				<0.01				<0.01				<0.01
H2	-0.01	0.15	0.417		-0.01	0.16	0.640		-0.01	0.18	0.464	
H3	-0.02	0.12	0.158		-0.01	0.12	0.724		-0.01	0.12	0.869	
Age	-0.29	0.01	<0.0001	0.05	-0.37	0.01	<0.0001		-0.18	0.01	<0.0001	0.03
Education				0.1				0.08				0.01
Secondary education	0.22	0.13	<0.0001		0.21	0.15	<0.0001		0.06	0.15	0.004	
Higher education	0.39	0.13	<0.0001		0.34	0.15	<0.0001		0.11	0.15	<0.0001	
Sex				<0.01				<0.01				0.01
Female	0.02	0.09	0.129		0.03	0.01	0.016		-0.01	0.11	0.732	
Employment				0.01								<0.01
Retired	0.04	0.13	0.020		0.08	0.12	<0.0001	0.02	0.01	0.14	0.744	
Other	-0.05	0.12	0.001		-0.06	0.14	<0.0001		-0.01	0.13	0.883	
Antidepressants				0.01				0.01				<0.01
Taking antidepressants	-0.05	0.21	0.001		-0.08	0.20	<0.0001		-0.02	0.22	0.104	
Smoking status				<0.01				0.01				<0.01
Past smoker	0.03	0.10	0.014		0.02	0.10	0.136		0.01	0.10	0.507	
Current smoker	-0.04	0.14	0.004		-0.05	0.17	0.002		-0.01	0.15	0.664	
Alcohol problem				<0.01				<0.01				<0.01
Yes	0.03	0.13	0.035		0.02	0.14	0.109		0.02	0.13	0.179	
Moderate physical activity				<0.01				<0.01				<0.01
<5 times/week	0.01	0.10	0.332		0.03	0.11	0.044		0.02	0.10	0.126	
Diabetes				<0.01				<0.01				<0.01
Yes	-0.04	0.18	0.005		-0.03	0.18	0.013		-0.04	0.20	0.006	
Systolic blood pressure	0.01	0.01	0.759	<0.01	0.05	0.01	0.042	<0.01	0.01	0.01	0.693	<0.01
Diastolic blood pressure	0.04	0.01	0.069	<0.01	-0.04	0.01	0.131	<0.01	0.01	0.01	0.839	<0.01
Baseline MOCA									0.60	0.02	<0.0001	0.38
**Overall model**	F (16, 5187) =82.87, p<0.0001, η2 =0.24				F (16, 4486) =66.41, p<0.0001, η2 =0.23				F (17, 2892) =111.73, p<0.0001, η2 =0.52			

Comparison group: hypertensive status = H1 (no hypertension), education = primary education, sex = male, employment = currently employed, antidepressant = not taking antidepressants, smoking status = non-smoker, alcohol problem = no alcohol problem, moderate physical activity = ≥5 times/week, diabetes = no diabetes.

Note: Hypertensive status consists of H1 (no hypertension), H2 (hypertension without medication), H3 (hypertensive with medication). Longitudinally, the dependent outcome (MoCA) at W3 was controlled for its baseline score.

Table 3. Multivariate regression results of the effects of hypertensive status (H1, H2, H3) on CTT-1 controlling for age, education and other socio-demographic and health behaviours (full model).

	Wave 1				Wave 3				Longitudinal			
**Source**	**β**	**SE**	**p-value**	**ηp2**	**β**	**SE**	**p-value**	**ηp2**	**β**	**SE**	**p-value**	**ηp2**
**CTT-1**												
Hypertensive status				<0.01				<0.01				<0.01
H2	0.02	1.19	0.174		-0.03	1.31	0.067		-0.02	1.50	0.202	
H3	0.02	0.96	0.265		-0.01	1.02	0.495		-0.01	1.09	0.811	
Age	0.47	0.08	<0.0001	0.13	0.46	0.09	<0.0001	0.12	0.22	0.09	<0.0001	0.03
Education				0.04				0.02				<0.01
Secondary education	-0.17	1.05	<0.0001		-0.13	1.21	<0.0001		-0.01	1.28	0.551	
Higher education	-0.23	1.03	<0.0001		-0.16	1.22	<0.0001		-0.02	1.34	0.472	
Sex				0.01				0.01				<0.01
Female	-0.08	0.72	<0.0001		-0.09	0.96	<0.0001		-0.04	1.18	0.044	
Employment				<0.01				0.01				<0.01
Retired	-0.06	1.13	0.004		-0.08	1.21	<0.0001		-0.03	1.35	0.139	
Other	0.01	0.83	0.272		0.04	1.49	0.028		0.01	1.35	0.701	
Antidepressants				0.01				0.01				<0.01
Taking antidepressants	0.07	1.88	<0.0001		0.08	2.16	<0.0001		0.03	3.56	0.270	
Smoking status				0.01				0.01				0.01
Past smoker	-0.04	0.75	0.001		-0.01	0.81	0.806		0.01	0.75	0.720	
Current smoker	0.04	1.02	0.004		0.07	1.89	<0.0001		0.08	2.64	0.003	
Alcohol problem				<0.01				<0.01				<0.01
Yes	0.03	0.88	0.008		0.02	1.43	0.250		-0.02	1.23	0.204	
Moderate physical activity				<0.01		0.87	0.584	<0.01				<0.01
<5 times/week	-0.01	0.73	0.416		-0.01				0.01	1.14	0.644	
Diabetes				<0.01				<0.01				<0.01
Yes	0.03	1.76	0.027		0.02	1.61	0.244		0.03	2.27	0.060	
Systolic blood pressure	-0.01	0.03	0.541	<0.01	0.02	0.04	0.499	<0.01	-0.01	0.04	0.648	<0.01
Diastolic blood pressure	-0.03	0.05	0.110	<0.01	0.01	0.06	0.726	<0.01	0.01	0.07	0.574	<0.01
Baseline CTT-1									0.51	0.03	<0.0001	0.26
**Overall model**	F (16, 5127) =69.19, p<0.0001, η2 =0.29				F (16, 4456) =66.99, p<0.0001, η2 =0.24				F (17, 2853) =122.03, p<0.0001, η2 =0.42			

Comparison group: hypertensive status = H1 (no hypertension), education = primary education, sex = male, employment = currently employed, antidepressant = not taking antidepressants, smoking status = non-smoker, alcohol problem = no alcohol problem, moderate physical activity = ≥5 times/week, diabetes = no diabetes.

Note: Hypertensive status consists of H1 (no hypertension), H2 (hypertension without medication), H3 (hypertensive with medication). Longitudinally, the dependent outcome (CTT-1) at W3 was controlled for its baseline score.

Table 4. Multivariate regression results of the effects of hypertensive status (H1, H2, H3) on CTT-2 controlling for age, education and other socio-demographic and health behaviours (full model).

	Wave 1				Wave 3				Longitudinal			
**Source**	**β**	**SE**	**p-value**	**ηp2**	**β**	**SE**	**p-value**	**ηp2**	**β**	**SE**	**p-value**	**ηp2**
**CTT-2**												
Hypertensive status				<0.01				<0.01				<0.01
H2	0.02	1.68	0.242		-0.01	1.95	0.990		0.01	1.94	0.917	
H3	0.02	1.42	0.129		0.01	1.49	0.948		0.02	1.36	0.323	
Age	0.47	0.10	<0.0001	0.13	0.46	0.10	<0.0001	0.13	0.17	0.11	<0.0001	0.03
Education				0.06				0.05				<0.01
Secondary education	-0.18	1.54	<0.0001		-0.19	1.76	<0.0001		-0.05	1.57	0.007	
Higher education	-0.27	1.51	<0.0001		-0.26	1.77	<0.0001		-0.07	1.56	<0.0001	
Sex				0.03				0.01				<0.01
Female	-0.05	1.07	<0.0001		-0.07	1.19	<0.0001		-0.02	1.03	0.156	
Employment				0.01				0.01				<0.01
Retired	-0.04	1.46	0.008		-0.19	1.47	0.006		0.03	1.40	0.114	
Other	0.04	1.34	0.004		-0.26	1.54	0.001		0.01	1.18	0.782	
Antidepressants				<0.01				0.01				<0.01
Taking antidepressants	0.05	2.52	0.001		0.07	2.22	<0.0001		0.03	2.27	0.010	
Smoking status				0.01				0.01				<0.01
Past smoker	-0.04	1.12	0.002		-0.02	1.16	0.135		-0.01	1.03	0.893	
Current smoker	0.33	1.52	0.008		0.03	1.88	0.021		0.01	1.47	0.562	
Alcohol problem				<0.01				<0.01				<0.01
Yes	0.02	1.44	0.038		-0.01	1.43	0.525		-0.02	1.32	0.031	
Moderate physical activity				<0.01				<0.01				<0.01
<5 times/week	0.02	1.15	0.049		0.01	1.28	0.365		0.01	1.06	0.465	
Diabetes				<0.01				<0.01				<0.01
Yes	0.03	2.42	0.065		0.03	2.31	0.023		0.03	2.30	0.031	
Systolic blood pressure	-0.17	0.05	0.453	<0.01	0.02	0.05	0.547	<0.01	-0.01	0.05	0.952	<0.01
Diastolic blood pressure	-0.03	0.76	0.085	<0.01	0.01	0.09	0.801	<0.01	-0.01	0.07	0.659	<0.01
Baseline CTT-2									0.67	0.03	<0.0001	0.48
**Overall model**	F (16, 5074) =95.01, p<0.0001, η2 =0.30				F (16, 4382) =95.01, p<0.0001, η2 =0.29				F (17, 2800) =281.11, p<0.0001, η2 =0.63			

Comparison group: hypertensive status = H1 (no hypertension), education = primary education, sex = male, employment = currently employed, antidepressant = not taking antidepressants, smoking status = non-smoker, alcohol problem = no alcohol problem, moderate physical activity = ≥5 times/week, diabetes = no diabetes.

Note: Hypertensive status consists of H1 (no hypertension), H2 (hypertension without medication), H3 (hypertensive with medication). Longitudinally, the dependent outcome (CTT-2) at W3 was controlled for its baseline score.

Table 5. Multivariate regression results of the effects of medication categories (BB, CB, ACE) on MMSE controlling for age, education and other socio-demographic and health behaviours (full model).

Source	Wave 1				Wave 3				Longitudinal			
	β	SE	p-value	ηp2	β	SE	p-value	ηp2	β	SE	p-value	ηp2
**MMSE**												
Medication categories				<0.01				<0.01				0.01
CB	-0.05	0.19	0.164		0.07	0.15	0.021		0.03	0.24	0.500	
ACE	-0.05	0.14	0.146		0.05	0.12	0.193		0.09	0.17	0.068	
Age	-0.35	0.01	<0.0001	0.07	-0.23	0.01	<0.0001	0.03	-0.08	0.01	<0.0001	0.05
Education				0.09				0.07				0.06
Secondary education	0.25	0.16	<0.0001		0.28	0.15	<0.0001		0.25	0.21	<0.0001	
Higher education	0.34	0.16	<0.0001		0.29	0.14	<0.0001		0.27	0.20	<0.0001	
Sex				<0.01				0.01				<0.01
Female	0.04	0.12	0.153		0.09	0.10	0.003		0.02	0.15	0.653	
Employment				<0.01				<0.01				<0.01
Retired	0.10	0.16	0.008		-0.01	0.12	0.909		-0.01	0.19	0.931	
Other	0.01	0.18	0.947		-0.05	0.15	0.184		-0.01	0.17	0.924	
Antidepressants				<0.01				0.01				<0.01
Taking antidepressants	-0.04	0.24	0.227		-0.11	0.22	0.003		-0.01	0.24	0.980	
Smoking status				0.01				<0.01				<0.01
Past smoker	0.02	0.12	0.453		0.05	0.10	0.108		0.02	0.15	0.606	
Current smoker	-0.06	0.20	0.066		-0.02	0.17	0.492		-0.02	0.25	0.656	
Alcohol problem				<0.01				<0.01				<0.01
Yes	0.03	0.95	0.341		0.05	0.13	0.078		0.01	0.20	0.761	
Moderate physical activity				<0.01				<0.01				<0.01
<5 times/week	0.04	0.12	0.126		0.05	0.11	0.045		-0.04	0.17	0.403	
Diabetes				<0.01				<0.01				<0.01
Yes	0.01	0.18	0.691		-0.01	0.15	0.618		-0.06	0.23	0.177	
Systolic blood pressure	0.06	0.01	0.214	<0.01	0.04	0.01	0.409	<0.01	-0.06	0.01	0.370	<0.01
Diastolic blood pressure	-0.02	0.01	0.605	<0.01	-0.02	0.01	0.660	<0.01	-0.06	0.01	0.389	<0.01
Baseline MMSE									0.33	0.06	<0.0001	0.11
**Overall model**	F (16, 1002 =19.78, p<0.0001, η2 =0.24				F (16, 1045) =14.38, p<0.0001, η2 =0.18				F (17, 491) =7.83, p<0.0001, η2 =0.27			

Comparison group: medication categories = BB (beta-blockers), education = primary education, sex = male, employment = currently employed, antidepressant = not taking antidepressants, smoking status = non-smoker, alcohol problem = no alcohol problem, moderate physical activity = ≥5 times/week, diabetes = no diabetes.

Note: Medication categories consists of BB (beta-blockers), CB (calcium-channel blockers), ACE (angiotensin converting enzyme inhibitors). Longitudinally, the dependent outcome (MMSE) at W3 was controlled for its baseline score.

Table 6. Multivariate regression results of the effects of medication categories (BB, CB, ACE) on MoCA controlling for age, education and other socio-demographic and health behaviours (full model).

Source	Wave 1				Wave 3				Longitudinal			
	β	SE	p-value	ηp2	β	SE	p-value	ηp2	β	SE	p-value	ηp2
**MOCA**												
Medication categories				<0.01				<0.01				<0.01
CB	-0.01	0.34	0.895		0.01	0.32	0.752		-0.02	-0.65	0.518	
ACE	-0.01	0.24	0.533		0.02	0.24	0.605		-0.04	-1.09	0.276	
Age	-0.33	0.02	<0.0001	0.07	-0.31	0.02	<0.0001	0.07	-0.04	-0.74	0.460	<0.01
Education				0.12				0.09				0.03
Secondary education	0.26	0.27	<0.0001		0.27	0.29	<0.0001		0.09	1.84	0.066	
Higher education	0.40	0.27	<0.0001		0.35	0.27	<0.0001		0.18	3.61	<0.0001	
Sex				<0.01				<0.01				<0.01
Female	-0.02	0.22	0.583		0.05	0.19	0.094		-0.03	-0.76	0.448	
Employment				0.01				0.01				0.01
Retired	0.07	0.28	0.056		0.06	0.28	0.062		-0.06	-1.39	0.166	
Other	-0.01	0.30	0.742		-0.04	0.28	0.189		0.01	0.29	0.774	
Antidepressants				<0.01				0.02				<0.01
Taking antidepressants	0.01	0.41	0.787		-0.12	0.41	<0.0001		-0.01	-0.44	0.657	
Smoking status				0.01				0.01				<0.01
Past smoker	0.02	0.22	0.585		-0.03	0.20	0.311		-0.05	-1.10	0.237	
Current smoker	-0.07	0.34	0.037		-0.09	0.33	0.002		-0.02	-0.49	0.622	
Alcohol problem				<0.01				0.01				0.01
Yes	0.01	0.32	0.643		0.09	0.26	0.001		0.05	1.63	0.104	
Moderate physical activity				<0.01				<0.01				<0.01
<5 times/week	0.02	0.23	0.463		0.04	0.24	0.130		0.03	0.95	0.342	
Diabetes				<0.01				<0.01				<0.01
Yes	-0.04	0.32	0.134		-0.02	0.30	0.410		-0.03	-0.65	0.515	
Systolic blood pressure	0.04	0.01	0.431	<0.01	0.03	0.01	0.509	<0.01	-0.10	-1.54	0.124	0.01
Diastolic blood pressure	-0.01	0.02	0.913	<0.01	-0.04	0.02	0.367	<0.01	0.11	1.70	0.075	0.01
Baseline MOCA									0.62	14.15	<0.0001	0.40
**Overall model**	F (16, 999) =23.06, p<0.0001, η2 =0.27				F (16, 1037) =21.87, p<0.0001, η2 =0.25				F (17, 445) =29.92, p<0.0001, η2 =0.53			

Comparison group: medication categories = BB (beta-blockers), education = primary education, sex = male, employment = currently employed, antidepressant = not taking antidepressants, smoking status = non-smoker, alcohol problem = no alcohol problem, moderate physical activity = ≥5 times/week, diabetes = no diabetes.

Note: Medication categories consists of BB (beta-blockers), CB (calcium-channel blockers), ACE (angiotensin converting enzyme inhibitors). Longitudinally, the dependent outcome (MoCA) at W3 was controlled for its baseline score.

Table 7. Multivariate regression results of the effects of medication categories (BB, CB, ACE) on CTT-1 controlling for age, education and other socio-demographic and health behaviours (full model).

	Wave 1				Wave 3				Longitudinal			
**Source**	**β**	**SE**	**p-value**	**ηp2**	**β**	**SE**	**p-value**	**ηp2**	**β**	**SE**	**p-value**	**ηp2**
**CTT-1**												
Medication categories				<0.01				<0.01				<0.01
CB	-0.02	2.60	0.568		-0.05	2.94	0.117		0.02	3.76	0.623	
ACE	-0.01	2.05	0.801		-0.05	2.25	0.169		0.02	1.96	0.542	
Age	0.44	0.15	<0.0001	0.12	0.46	0.20	<0.0001	0.14	0.19	0.26	0.005	0.03
Education				0.04				0.03				0.01
Secondary education	-0.13	2.18	<0.0001		-0.16	2.54	<0.0001		-0.09	2.61	0.039	
Higher education	-0.20	2.23	<0.0001		-0.17	2.39	<0.0001		-0.09	2.58	0.031	
Sex				<0.01				0.01				<0.01
Female	-0.03	1.67	0.319		-0.10	1.69	<0.0001		-0.03	2.15	0.410	
Employment				<0.01				0.01				<0.01
Retired	-0.01	2.06	0.814		-0.09	2.78	0.045		-0.05	3.02	0.337	
Other	0.05	2.20	0.109		0.03	2.53	0.310		0.01	2.68	0.954	
Antidepressants				<0.01				0.01				<0.01
Taking antidepressants	0.27	3.73	0.389		0.08	4.39	0.033		-0.03	3.74	0.295	
Smoking status				0.01				0.01				<0.01
Past smoker	-0.06	1.71	0.033		0.01	1.73	0.817		0.04	2.00	0.279	
Current smoker	0.06	2.79	0.079		0.09	3.37	0.006		0.04	4.56	0.467	
Alcohol problem				<0.01				0.01				<0.01
Yes	0.05	2.33	0.076		-0.06	2.07	0.003		-0.02	2.85	0.572	
Moderate physical activity				<0.01				<0.01				<0.01
<5 times/week	-0.03	1.84	0.238		0.02	1.98	0.459		-0.01	2.20	0.893	
Diabetes				<0.01				<0.01				<0.01
Yes	0.05	11.97	0.165		0.02	2.64	0.512		-0.01	4.51	0.913	
Systolic blood pressure	0.04	0.77	0.408	<0.01	0.06	1.73	0.242	<0.01	0.05	0.10	0.419	<0.01
Diastolic blood pressure	-0.07	0.12	0.082	<0.01	-0.01	3.37	0.860	<0.01	-0.09	0.15	0.086	0.01
Baseline CTT-1									0.58	0.08	<0.0001	0.33
**Overall model**	F (16, 983) =26.20, p<0.0001, η2 =0.30				F (16, 1029) =25.97, p<0.0001, η2 =0.29				F (17, 441) =28.10, p<0.0001, η2 =0.52			

Comparison group: medication categories = BB (beta-blockers), education = primary education, sex = male, employment = currently employed, antidepressant = not taking antidepressants, smoking status = non-smoker, alcohol problem = no alcohol problem, moderate physical activity = ≥5 times/week, diabetes = no diabetes.

Note: Medication categories consists of BB (beta-blockers), CB (calcium-channel blockers), ACE (angiotensin converting enzyme inhibitors). Longitudinally, the dependent outcome (CTT-1) at W3 was controlled for its baseline score.

Table 8. Multivariate regression results of the effects of medication categories (BB, CB, ACE) on CTT-2 controlling for age, education and other socio-demographic and health behaviours (full model).

	Wave 1				Wave 3				Longitudinal			
**Source**	**β**	**SE**	**p-value**	**ηp2**	**β**	**SE**	**p-value**	**ηp2**	**β**	**SE**	**p-value**	**ηp2**
**CTT-2**												
Medication categories				<0.01				<0.01				<0.01
CB	-0.04	4.14	0.163		-0.03	3.85	0.396		-0.01	4.15	0.934	
ACE	0.01	3.17	0.937		-0.03	3.07	0.328		0.02	2.69	0.519	
Age	0.47	0.26	<0.0001	0.14	0.40	0.23	<0.0001	0.11	0.72	0.25	<0.0001	<0.01
Education				0.05				0.06				0.03
Secondary education	-0.17	3.51	<0.0001		-0.21	3.64	<0.0001		-0.12	3.14	0.002	
Higher education	-0.24	3.54	<0.0001		-0.27	3.54	<0.0001		-0.14	3.37	0.001	
Sex				<0.01				0.01				<0.01
Female	-0.02	2.73	0.525		-0.08	2.63	0.009		0.01	2.51	0.823	
Employment				<0.01				<0.01				0.02
Retired	-0.03	3.46	0.364		-0.02	3.48	0.592		0.07	3.10	0.066	
Other	0.01	3.64	0.745	<0.01	0.05	4.07	0.195		0.09	3.61	0.019	
Antidepressants								<0.01				<0.01
Taking antidepressants	0.03	6.30	0.319		0.04	5.33	0.181		0.01	5.24	0.991	
Smoking status				<0.01				<0.01				<0.01
Past smoker	-0.02	2.89	0.412		-0.01	2.57	0.732		-0.02	2.48	0.508	
Current smoker	0.03	4.05	0.319		0.03	4.43	0.288		-0.01	4.50	0.727	
Alcohol problem				<0.01				0.01				<0.01
Yes	0.05	4.28	0.068		-0.06	3.12	0.009		-0.04	3.51	0.174	
Moderate physical activity				<0.01				<0.01				<0.01
<5 times/week	0.02	3.15	0.500		0.04	3.21	0.161		-0.01	2.74	0.625	
Diabetes				<0.01				<0.01				<0.01
Yes	-0.01	4.60	0.965		-0.01	4.14	0.878		-0.01	4.24	0.654	
Systolic blood pressure	0.02	0.11	0.650	<0.01	0.05	0.11	0.232	<0.01	0.02	0.11	0.752	<0.01
Diastolic blood pressure	-0.05	0.18	0.176	<0.01	-0.04	0.18	0.401	<0.01	-0.06	0.17	0.179	<0.01
Baseline CTT-2									0.72	0.06	<0.0001	0.53
**Overall model**	F (16, 972) =27.45, p<0.0001, η2 =0.31				F (16, 1009) =24.53, p<0.0001, η2 =0.28				F (17, 433) =49.77, p<0.0001, η2 =0.66			

Comparison group: medication categories = BB (beta-blockers), education = primary education, sex = male, employment = currently employed, antidepressant = not taking antidepressants, smoking status = non-smoker, alcohol problem = no alcohol problem, moderate physical activity = ≥5 times/week, diabetes = no diabetes.

Note: Medication categories consists of BB (beta-blockers), CB (calcium-channel blockers), ACE (angiotensin converting enzyme inhibitors). Longitudinally, the dependent outcome (CTT-2) at W3 was controlled for its baseline score.

Table 9. Multivariate regression results of the effects of medication status (monotherapy and combination therapy) on MMSE controlling for age, education and other socio-demographic and health behaviours (full model).

Source	Wave 1				Wave 3				Longitudinal			
	**β**	**SE**	**p-value**	**ηp2**	**β**	**SE**	**p-value**	**ηp2**	**β**	**SE**	**p-value**	**ηp2**
**MMSE**												
Medication status				<0.01				<0.01				<0.01
Combination therapy	0.02	0.10	0.318		-0.01	-0.01	0.968		-0.01	0.09	0.921	
Age	-0.31	0.01	<0.0001	0.06	-0.28	-0.06	<0.0001	0.05	-0.24	0.01	<0.0001	0.04
Education				0.09				0.05				0.02
Secondary education	0.24	0.12	<0.0001		0.22	0.11	<0.0001	0.01	0.09	0.15	0.021	
Higher education	0.33	0.12	<0.0001		0.25	0.11	<0.0001		0.13	0.15	<0.0001	
Sex				<0.01				0.01				<0.01
Female	0.04	0.10	0.101		0.08	0.08	<0.0001		-0.01	0.09	0.988	
Employment				<0.01				<0.01				<0.01
Retired	0.08	0.14	0.007		0.01	0.10	0.619		0.05	0.13	0.132	
Other	0.02	0.14	0.548		-0.05	0.12	0.050		0.02	0.12	0.538	
Antidepressants				0.01				0.01				<0.01
Taking antidepressants	-0.06	0.21	0.020		-0.08	0.16	0.002		-0.01	0.17	0.888	
Smoking status				0.01				<0.01				<0.01
Past smoker	0.03	0.10	0.125		0.03	0.08	0.210		-0.01	0.10	0.914	
Current smoker	-0.05	0.16	0.044		-0.03	0.14	0.183		-0.03	0.17	0.334	
Alcohol problem				<0.01				<0.01				<0.01
Yes	0.02	0.15	0.400		0.03	0.12	0.179		-0.01	0.12	0.823	
Moderate physical activity				<0.01				<0.01				<0.01
<5 times/week	0.01	0.12	0.564		0.05	0.09	0.005		-0.01	0.11	0.986	
Diabetes				<0.01				<0.01				<0.01
Yes	-0.04	0.18	0.191		-0.01	0.11	0.705		-0.03	0.13	0.170	
Systolic blood pressure	0.06	0.01	0.053	<0.01	0.06	0.08	0.078	<0.01	-0.02	0.01	0.515	<0.01
Diastolic blood pressure	-0.03	0.01	0.359	<0.01	-0.04	0.14	0.223	<0.01	0.05	0.01	0.138	<0.01
Baseline MMSE									0.34	0.06	<0.0001	0.15
**Overall model**	F (15, 1872 =32.99, p<0.0001, η2 =0.21				F (15, 1847) =23.72, p<0.0001, η2 =0.16				F (16, 1373) =34.31, p<0.0001, η2 =0.29			

Comparison group: medication status = Monotherapy, education = primary education, sex = male, employment = currently employed, antidepressant = not taking antidepressants, smoking status = non-smoker, alcohol problem = no alcohol problem, moderate physical activity = ≥5 times/week, diabetes = no diabetes. Note: Medication status consists of monotherapy and combination therapy. Longitudinally, the dependent outcome (MMSE) at W3 was controlled for its baseline score.

Table 10. Multivariate regression results of the effects of medication status (monotherapy and combination therapy) on MoCA controlling for age, education and other socio-demographic and health behaviours (full model).

	Wave 1				Wave 3				Longitudinal			
**Source**	**β**	**SE**	**p-value**	**ηp2**	**β**	**SE**	**p-value**	**ηp2**	**β**	**SE**	**p-value**	**ηp2**
**MOCA**												
Medication status				<0.01				<0.01				<0.01
Combination therapy	0.04	0.16	0.039		0.04	0.29	0.084		0.03	0.16	0.204	
Age	-0.36	0.01	<0.0001	0.08	-0.36	-0.15	<0.0001	0.09	-0.20	0.01	<0.0001	0.04
Education				0.10				0.07				0.01
Secondary education	0.22	0.20	<0.0001		0.20	0.22	<0.0001		0.04	0.22	0.186	
Higher education	0.35	0.20	<0.0001		0.30	0.21	<0.0001		0.09	0.22	0.001	
Sex				<0.01				<0.01				<0.01
Female	-0.01	0.17	0.676		0.26	0.16	0.236		-0.02	0.17	0.372	
Employment				0.01				0.01				<0.01
Retired	0.10	0.22	0.001		0.09	0.20	0.001		0.02	0.21	0.471	
Other	-0.01	0.23	0.960		-0.22	0.23	0.377		0.03	0.22	0.208	
Antidepressants				<0.01				0.02				<0.01
Taking antidepressants	-0.05	0.33	0.033		-0.11	0.31	<0.0001		-0.01	0.30	0.612	
Smoking status				0.01				<0.01				<0.01
Past smoker	0.04	0.17	0.063		0.01	0.16	0.817		-0.01	0.17	0.706	
Current smoker	-0.04	0.27	0.103		-0.06	0.30	0.021		-0.02	0.28	0.319	
Alcohol problem				<0.01				<0.01				<0.01
Yes	0.01	0.24	0.588		0.04	0.24	0.035		0.03	0.22	0.142	
Moderate physical activity				<0.01				<0.01				<0.01
<5 times/week	0.03	0.18	0.108		0.03	0.19	0.177		0.03	0.18	0.374	
Diabetes				<0.01				<0.01				0.01
Yes	-0.04	0.22	0.036		-0.05	0.22	0.035		-0.05	0.24	0.008	
Systolic blood pressure	0.04	0.01	0.214	<0.01	0.07	0.01	0.037	<0.01	0.02	0.01	0.499	<0.01
Diastolic blood pressure	-0.01	0.01	0.970	<0.01	-0.04	0.01	0.189	<0.01	-0.02	0.01	0.523	<0.01
Baseline MOCA									0.62	0.03	<0.0001	0.41
**Overall model**	F (15, 1866) =45.24, p<0.0001, η2 =0.27				F (15, 1828) =36.77, p<0.0001, η2 =0.23				F (16, 1235) =91.83, p<0.0001, η2 =0.54			

Comparison group: medication status = Monotherapy, education = primary education, sex = male, employment = currently employed, antidepressant = not taking antidepressants, smoking status = non-smoker, alcohol problem = no alcohol problem, moderate physical activity = ≥5 times/week, diabetes = no diabetes. Note: Medication status consists of monotherapy and combination therapy. Longitudinally, the dependent outcome (MoCA) at W3 was controlled for its baseline score.

Table 11. Multivariate regression results of the effects of medication status (monotherapy and combination therapy) on CTT-1 controlling for age, education and other socio-demographic and health behaviours (full model).

	Wave 1				Wave 3				Longitudinal			
**Source**	**β**	**SE**	**p-value**	**ηp2**	**β**	**SE**	**p-value**	**ηp2**	**β**	**SE**	**p-value**	**ηp2**
**CTT-1**												
Medication status				<0.01				<0.01				<0.01
Combination therapy	-0.02	1.36	0.289		-0.02	1.41	0.320		-0.03	1.42	0.102	
Age	0.49	0.11	<0.0001	0.15	0.50	0.13	<0.0001	0.17	0.26	0.14	<0.0001	0.06
Education				0.04				0.03				<0.01
Secondary education	-0.15	1.73	<0.0001		-0.15	1.83	<0.0001		-0.03	1.87	0.237	
Higher education	-0.21	1.75	<0.0001		-0.17	1.84	<0.0001		-0.02	1.93	0.393	
Sex				<0.01				0.01				<0.01
Female	-0.06	1.38	0.003		-0.11	1.30	<0.0001		-0.05	1.43	0.028	
Employment				0.01				0.02				<0.01
Retired	-0.05	1.73	0.044		-0.09	2.01	0.002		-0.05	1.97	0.085	
Other	0.04	1.76	0.079		0.04	1.92	0.108		-0.01	1.87	0.980	
Antidepressants				0.01				0.01				<0.01
Taking antidepressants	0.06	3.17	0.018		0.08	2.81	0.002		-0.01	2.38	0.678	
Smoking status				0.01				0.01				0.01
Past smoker	-0.05	1.44	0.031		-0.01	1.36	0.616		0.01	1.38	0.988	
Current smoker	0.50	2.18	0.020		0.08	2.69	0.002		0.08	3.46	0.014	
Alcohol problem				<0.01				<0.01				<0.01
Yes	0.39	1.81	0.022		0.01	2.21	0.601		-0.01	1.80	0.577	
Moderate physical activity				<0.01				<0.01				<0.01
<5 times/week	-0.02	1.47	0.293		-0.02	1.48	0.334		-0.01	1.66	0.692	
Diabetes				<0.01				<0.01				<0.01
Yes	0.05	2.25	0.047		0.03	1.95	0.226		0.04	2.64	0.126	
Systolic blood pressure	-0.02	0.05	0.410	0.01	0.01	0.06	0.841	<0.01	-0.01	0.06	0.711	<0.01
Diastolic blood pressure	-0.01	0.09	0.651	<0.01	0.02	0.09	0.634	<0.01	0.01	0.10	0.689	<0.01
Baseline CTT-1									0.55	0.05	<0.0001	0.31
**Overall model**	F (15, 1825) =52.83, p<0.0001, η2 =0.30				F (15, 1813) =48.50, p<0.0001, η2 =0.29				F (16, 1206) =78.79, p<0.0001, η2 =0.51			

Comparison group: medication status = Monotherapy, education = primary education, sex = male, employment = currently employed, antidepressant = not taking antidepressants, smoking status = non-smoker, alcohol problem = no alcohol problem, moderate physical activity = ≥5 times/week, diabetes = no diabetes. Note: Medication status consists of monotherapy and combination therapy. Longitudinally, the dependent outcome (CTT-1) at W3 was controlled for its baseline score.

Table 12. Multivariate regression results of the effects of medication status (monotherapy and combination therapy) on CTT-2 controlling for age, education and other socio-demographic and health behaviours (full model).

	**Wave 1**				**Wave 3**				**Longitudinal**			
**Source**	**β**	**SE**	**p-value**	**ηp2**	**β**	**SE**	**p-value**	**ηp2**	**β**	**SE**	**p-value**	**ηp2**
**CTT-2**												
Medication status				<0.01				<0.01				<0.01
Combination therapy	-0.02	2.04	0.442		-0.01	1.96	0.666		-0.03	1.56	0.052	
Age	0.51	0.17	<0.0001	0.16	0.44	0.16	<0.0001	0.13	0.15	0.15	<0.0001	0.03
Education				0.05				0.07				0.01
Secondary education	-0.15	2.58	<0.0001		-0.21	2.73	<0.0001		-0.06	2.21	0.008	
Higher education	-0.23	2.57	<0.0001		-0.29	2.66	<0.0001		-0.08	2.24	0.001	
Sex				<0.01				0.01				0.01
Female	-0.03	2.04	0.148		0.08	2.02	<0.0001		-0.02	1.74	0.213	
Employment				0.01				0.01				<0.01
Retired	-0.06	2.59	0.020		-0.04	2.73	0.141		0.03	2.14	0.294	
Other	0.02	2.75	0.374		0.04	3.02	0.163		0.01	2.20	0.525	
Antidepressants				<0.01				0.01				<0.01
Taking antidepressants	0.04	4.23	0.050		0.07	3.50	0.002		0.02	2.99	0.169	
Smoking status				0.01				<0.01				<0.01
Past smoker	-0.03	2.18	0.166		-0.02	1.98	0.422		-0.01	1.72	0.558	
Current smoker	0.04	3.13	0.034		0.03	3.46	0.182		0.02	2.80	0.319	
Alcohol problem				<0.01				<0.01				<0.01
Yes	0.04	3.04	0.028		-0.02	2.42	0.220		-0.02	2.37	0.187	
Moderate physical activity				<0.01				<0.01				<0.01
<5 times/week	0.02	2.38	0.348		0.02	2.36	0.359		-0.02	1.79	0.340	
Diabetes				<0.01				<0.01				<0.01
Yes	0.04	3.11	0.071		0.04	2.87	0.077		0.04	2.68	0.091	
Systolic blood pressure	-0.03	0.08	0.321	<0.01	0.01	0.08	0.801	<0.01	0.02	0.07	0.485	<0.01
Diastolic blood pressure	-0.01	0.13	0.702	<0.01	0.02	0.13	0.631	<0.01	-0.01	0.11	0.713	<0.01
Baseline CTT-2									0.68	0.04	<0.0001	0.50
**Overall model**	F (15, 1794) =55.03, p<0.0001, η2 =0.32				F (15, 1766) =47.46, p<0.0001, η2 =0.29				F (16, 1168) =130.23, p<0.0001, η2 =0.64			

Comparison group: medication status = Monotherapy, education = primary education, sex = male, employment = currently employed, antidepressant = not taking antidepressants, smoking status = non-smoker, alcohol problem = no alcohol problem, moderate physical activity = ≥5 times/week, diabetes = no diabetes. Note: Medication status consists of monotherapy and combination therapy. Longitudinally, the dependent outcome (CTT-2) at W3 was controlled for its baseline score.
